# ASSESSING SYMPTOM NETWORKS OF DEPRESSIVE SYMPTOMS IN OLDER ADULTS: A CROSS-NATIONAL POPULATION-BASED STUDY

**DOI:** 10.1093/geroni/igad104.2433

**Published:** 2023-12-21

## Abstract

**Background:**

The complex relationships across depressive symptoms in older adults may differ across countries. We aimed to generate symptom networks of depressive symptoms in older adults and explore different patterns of depressive symptoms across countries from a mechanism perspective.

**Methods:**

We used harmonized data on 126,025 individuals ≥65 yrs from the Health and Retirement Study (HRS) family data from US, Mexico, UK, China, Israel, European countries, and Korea. We constructed dynamic networks with eight depressive symptoms using cross-lagged panel network analysis.

**Results:**

We identified two patterns in the networks of depressive symptoms: sadness-centered pattern and non-sadness-centered pattern. In the sadness-centered pattern, “felt sad” was the strongest predictor of other depressive symptoms in the United State (rs=4.38), Mexico (rs=4.96), and European and Israel (rs=4.61). Moreover, “felt depression” was found to have strong relationship with “felt sad” (r=0.586, 0.599, and 0.598, respectively). In the non-sadness-centered pattern, “bothered by little things” had the highest node strength in England (rs=8.16) and “could not get going” had the highest node strength in Korea (rs=10.18).

**Discussion:**

Our study demonstrated two patterns in the networks of depressive symptoms in older adults. In the sadness-centered pattern, a combined intervention for analyzing the cause of sadness and alleviating it should be considered. Keywords: Global health; Depression; Older adults; Symptom network

